# Supplementary material for: Men’s late presentation for HIV care in Eastern Uganda: The role of masculinity norms
Source: PLoS One. 2022 Nov 17;17(11):e0277534. doi: 10.1371/journal.pone.0277534 (PMC9671417; doi:10.1371/journal.pone.0277534)
Supplement: S1 File — (DOCX) [file pone.0277534.s001.docx]

**FOCUS GROUP DISCUSSION GUIDE**

**Facilitator’s Welcome, Introduction, Purpose of FGD and ground rules to participants:** Welcome and thank you for volunteering to take part in this focus group discussion. You have been asked to participate, as your point of view is important. We understand you are busy and we appreciate your time.

**Introductions and purpose of the study**: We are from Makerere University. We want to thank you for joining us. The aim of the discussion is to explore the role of masculinity norms in men’s presentation for HIV care in Jinja district. You have been selected to represent such views. So, when discussing the questions, you should be making reference to what happens in the community and not necessarily your own personal experiences. Before we get started we are going to pass out this consent form. This provides information about what we will be doing today in the discussion group. It also gives you a chance to decide if you are willing to take part in today’s group or not. The discussion will take no more than two hours and will be taped but all the information will be confidential.

**Ground rules:**

- The most important rule is that only one person speaks at a time. There may be a temptation to jump in when someone is talking but please wait until they have finished.
- There are no right or wrong answers. You do not have to speak in any particular order.
- When you do have something to say, please feel free to do so. There are many of you in the group and it is important that I obtain the views of each of you. You do not have to agree with the views of other people in the group.
- Please, refrain from discussing the comments of other group members outside the focus group.

Does anyone have any questions? (Answers). OK, let us begin

**FOCUS GROUP DISCUSSION GUIDE**

**TOPIC:** TO EXPLORE THE ROLE OF MASCULINITY NORMS IN MEN’S LATE PRESENTATION FOR HIV CARE IN EASTERN UGANDA, JINJA DISTRICT

**Preliminary section**

FGD date: ……………………… Language used: ……………………..

Moderator: …………………………… FGD category: …………………….

Note taker: ……………………………. FGD venue: …………………………

Time started: ………………………… Time ended: ………………………..

| **Masculinity norms**  1. Let’s talk about what it means to be a man in this region? (*Probe: What are the things that a man has to do to be considered a man in this region/district?* *Under which circumstances are men encouraged to prove themselves, or forced to prove themselves?)*  2. What are some of the most desirable masculine traits in your community?  3. Are all men able to live up to these expectations? *(Probe: Which factors facilitate their achievement of these expectations? Which factors compromise their achievement of these expectations? When do men feel inadequately masculine? When do men feel sufficiently masculine?)*  4. What kind of men are not viewed as masculine? *(Probe: what are their characteristics, occupation, education level, social economic status?)*  **Masculinity norms, HIV and presentation into care**  5. Now, let’s talk about what it means to be a man and being HIV positive. How is HIV/AIDs perceived in this region? Do you think being HIV positive changes how a man is viewed in this region? *(Probe: answers with examples (By his friends, his family, himself if yes, how?)*  6. How do the beliefs and definition of what it means to be a man in this region influence men’s presentation into HIV care? *(Probe: is the influence positive/negative? do men find it easy to seek HIV treatment, do they find it difficult to start HIV treatment?)*  7. Why do some men appear to have greater difficulties or less motivation to present early into HIV care and how do others find it easy to do so? (*Probe: Are there aspects of masculinity that make it easy for men to seek help? which ones? Why? Are there aspects of masculinity that make it difficult for men to seek help? Which ones? Why?*)  8. In your opinion, how do you think HIV positive men can be encouraged/influenced to present early into HIV care while preserving masculinity? *(Probe: with examples, who is the biggest influence of men in this community? Is it family members, friends, media? What channels can be used, what messages should be designed when it comes to influencing men to present early into HIV care?)*  9. Is there any other issue related to this topic that we did not cover that you would like to discuss?  ***Thank you for your time and the input that you gave*** |  |
| --- | --- |
