# Supplementary material for: Men’s late presentation for HIV care in Eastern Uganda: The role of masculinity norms
Source: PLoS One. 2022 Nov 17;17(11):e0277534. doi: 10.1371/journal.pone.0277534 (PMC9671417; doi:10.1371/journal.pone.0277534)
Supplement: S2 File — (DOCX) [file pone.0277534.s002.docx]

**INDEPTH INTERVIEW GUIDE**

**TOPIC:** TO EXPLORE THE ROLE OF MASCULINITY NORMS IN MEN’S LATE PRESENTATION FOR HIV CARE IN EASTERN UGANDA, JINJA DISTRICT

Participant number……………

Name of interviewer………………………… Date of interview……...............................

**Socio-demographic characteristics**

| **SN** | **Question** | **Codes** |
| --- | --- | --- |
| 101 | How old are you? | Age……………  (completed years) |
| 102 | Which year were you born? | ……./……./………..  dd/mm/yyyy |
| 103 | What is your current marital status? | Single……………1  Married………….2  Separated………...3  Widowed………...4 |
| 104 | What is your religion? | Catholic…………..1  Anglican………….2  Muslim……………3  Pentecostal………..4  Others (specify)…...5 |
| 105 | What is your highest level of education? | None………………1  Primary……………2  Secondary…………3  Tertiary……………4  Others(specify)……5 |
| 106 | What is your occupation? |  |

| **Masculinity norms**  1. Let’s talk about what it means to be a man in this region? (*Probe: What are the things that a man has to do to be considered a man in this region/district?* *Under which circumstances are men encouraged to prove themselves, or forced to prove themselves?)*  2. What are some of the most desirable masculine traits in your community?  3. Are all men able to live up to these expectations? *(Probe: Which factors facilitate their achievement of these expectations? Which factors compromise their achievement of these expectations? When do men feel inadequately masculine? When do men feel sufficiently masculine?)*  4. What kind of men are not viewed as masculine? *(Probe: what are their characteristics, occupation, education level, social economic status?)*  5. How about you? What does being a man mean to you? (*Probe: how would you define a masculine man?) What are the healthy masculine traits, how can we promote them? What are the negative masculine traits?)*  **Masculinity norms, HIV and presentation into care**  6. Now, let’s talk about what it means to be a man and being HIV positive. How is HIV/AIDs perceived in this region? Do you think being HIV positive changes how a man is viewed in this region? *(Probe: answers with examples (By his friends, his family, himself if yes, how?)*  7. Did being HIV positive change how you were viewed/seen as a man? *(Probe: by the society, your friends, family if yes, how?)*  8. How do the beliefs and definition of what it means to be a man in this region influence men’s presentation into HIV care? *(Probe: is the influence positive/negative? do men find it easy to seek HIV treatment, do they find it difficult to start HIV treatment?)*  9. How about you? Did your beliefs of what it means to be a man influence your access/presentation into HIV care? (*Probe: how did they influence your decision to present into HIV care, was it easy/difficult for you? Explain/How?*)  10. Why do some men appear to have greater difficulties or less motivation to present early into HIV care and how do others find it easy to do so? (*Probe: Are there aspects of masculinity that make it easy for men to seek help? which ones? Why? Are there aspects of masculinity that make it difficult for men to seek help? Which ones? Why?*)  11. In your opinion, how do you think HIV positive men can be encouraged/influenced to present early into HIV care while preserving masculinity? *(Probe: with examples, who is the biggest influence of men in this community? Is it family members, friends, media? What channels can be used, what messages should be designed when it comes to influencing men to present early into HIV care?)*  12. Is there any other issue related to this topic that we did not cover that you would like to discuss?  ***Thank you for your time and the input that you gave*** |  |
| --- | --- |
