# Supplementary material for: Men’s late presentation for HIV care in Eastern Uganda: The role of masculinity norms
Source: PLoS One. 2022 Nov 17;17(11):e0277534. doi: 10.1371/journal.pone.0277534 (PMC9671417; doi:10.1371/journal.pone.0277534)
Supplement: Attachment — Submitted filename: Overall comments to authors.docx [file pone.0277534.s004.docx]

**Overall comments to authors:**

The use terms “HIV positive men” is stigmatising by WHO, please change and use terms like “men living with HIV” or “people living with HIV” or “patients living with HIV” throughout the manuscript.

Lines 75-76: “Although women in most parts of Sub-Saharan Africa (SSA), and Uganda in particular, have a higher prevalence of HIV than men”

Please use this source to support it:

- B Sartorius, JD VanderHeide, M Yang, EA Goosmann, et al. [Subnational mapping of HIV incidence and mortality among individuals aged 15–49 years in sub-Saharan Africa, 2000–18: a modelling study](https://scholar.google.com.au/citations?view_op=view_citation&hl=en&user=K7SUZ1YAAAAJ&citation_for_view=K7SUZ1YAAAAJ:L8Ckcad2t8MC). The Lancet HIV 8 (6), e363-e375. <https://www.sciencedirect.com/science/article/pii/S2352301821000515>

Lines 80-81: “Fewer men seek testing and counselling services on a routine basis leading to late diagnosis in late-stage disease progression”

Please use the following sources to support it:

- [The intention of men who have sex with men to participate in voluntary counseling and HIV testing and access free condoms in Indonesia](https://scholar.google.com.au/citations?view_op=view_citation&hl=en&user=K7SUZ1YAAAAJ&citation_for_view=K7SUZ1YAAAAJ:WF5omc3nYNoC). American Journal of Men's Health 12 (5), 1175-1184. <https://journals.sagepub.com/doi/full/10.1177/1557988318779737>
- [Perceptions among transgender women of factors associated with the access to HIV/AIDS-related health services in Yogyakarta, Indonesia](https://scholar.google.com.au/citations?view_op=view_citation&hl=en&user=K7SUZ1YAAAAJ&citation_for_view=K7SUZ1YAAAAJ:UebtZRa9Y70C). PloS one 14 (8), e0221013. <https://journals.plos.org/plosone/article?id=10.1371/journal.pone.0221013>
- [Risk Factors and the Impact of HIV among Women Living with HIV and Their Families in Yogyakarta and Belu District, Indonesia](https://scholar.google.com.au/citations?view_op=view_citation&hl=en&user=K7SUZ1YAAAAJ&cstart=20&pagesize=80&citation_for_view=K7SUZ1YAAAAJ:k_IJM867U9cC). ( see Pages 35-54: scoping review on barriers and facilitators of the access of men living with HIV to HIV services; pages 224-247: findings on barriers and facilitators of the access of men living with HIV to HIV services). <https://flex.flinders.edu.au/file/eac1a04e-a24b-4888-ace8-50f06805069e/1/Fauk%20Thesis2022.pdf>

Lines 82-83: Due to late presentation, men develop opportunistic infections with poor treatment outcomes [7] and high healthcare costs [REF].

Please use the following source to support ‘high healthcare costs:

- [Risk Factors and the Impact of HIV among Women Living with HIV and Their Families in Yogyakarta and Belu District, Indonesia](https://scholar.google.com.au/citations?view_op=view_citation&hl=en&user=K7SUZ1YAAAAJ&cstart=20&pagesize=80&citation_for_view=K7SUZ1YAAAAJ:k_IJM867U9cC). ( see Pages 35-54: scoping review on barriers and facilitators of the access of men living with HIV to HIV services; pages 224-247: findings on barriers and facilitators of the access of men living with HIV to HIV services). <https://flex.flinders.edu.au/file/eac1a04e-a24b-4888-ace8-50f06805069e/1/Fauk%20Thesis2022.pdf>

***Data management and analysis***

Are the field researchers (research assistants) the authors in the paper? If yes, please put their initials to make clear who did what. Who are the three people in the analysis team? Please provide their initials in bracket.

I suggest the authors use the following resources to support their description of data analysis:

- Cultural and religious determinants of HIV transmission: A qualitative study with people living with HIV in Belu and Yogyakarta, Indonesia. PLoS ONE 16(11): e0257906. <https://journals.plos.org/plosone/article?id=10.1371/journal.pone.0257906>
- Qualitative Data Analysis for Applied Policy Research. In: Bryman A, Burgess RG, editors. London: Routledge; 1994. p. 173–94.

**Discussion**

Lines 389-390: “Internalised stigma has been found to impact certain decisions like avoiding to seek care when needed.”

Please use the following resources to support it:

- HIV Stigma and Moral Judgement: Qualitative Exploration of the Experiences of HIV Stigma and Discrimination among Married Men Living with HIV in Yogyakarta. Int. J. Environ. Res. Public Health **2020**, 17, 636. <https://doi.org/10.3390/ijerph17020636>.
- [HIV stigma and discrimination: perspectives and personal experiences of healthcare providers in Yogyakarta and Belu, Indonesia](https://scholar.google.com.au/citations?view_op=view_citation&hl=en&user=K7SUZ1YAAAAJ&citation_for_view=K7SUZ1YAAAAJ:4DMP91E08xMC). *Frontiers in medicine* 8, 625787. <https://www.frontiersin.org/articles/10.3389/fmed.2021.625787/full>

Lines 394-396: “On the other hand, “enacted stigma,” which is the lived experience of discrimination against an HIV positive person through others’ actions or words, is also known to have a negative impact on health outcomes.”

Please use the following resources to support it:

- HIV Stigma and Moral Judgement: Qualitative Exploration of the Experiences of HIV Stigma and Discrimination among Married Men Living with HIV in Yogyakarta. Int. J. Environ. Res. Public Health **2020**, 17, 636. <https://doi.org/10.3390/ijerph17020636>.
- Stigma and Discrimination towards People Living with HIV in the Context of Families, Communities, and Healthcare Settings: A Qualitative Study in Indonesia. Int. J. Environ. Res. Public Health **2021**, 18, 5424. <https://doi.org/10.3390/ijerph18105424>
