# Supplementary material for: Men’s late presentation for HIV care in Eastern Uganda: The role of masculinity norms
Source: PLoS One. 2022 Nov 17;17(11):e0277534. doi: 10.1371/journal.pone.0277534 (PMC9671417; doi:10.1371/journal.pone.0277534)
Supplement: Attachment — Submitted filename: Response to Reviewers.docx [file pone.0277534.s005.docx]

The Editorial Team

PLOS ONE Journal

20^th^ October 2022

Dear Editor,

We express our cordial gratitude for the valuable and constructive review of our paper.

The reviewers’ comments have helped us to further strengthen the overall quality of the paper. With utmost care, we have incorporated all the suggestions/corrections as proposed by the reviewer.

The specific responses to the reviewer’s comments are listed below.

|  | **COMMENTS** | **Authors’ RESPONSE**  **to COMMENTS** | **Indicator/Location of**  **Response in the ‘Manuscript’** |
| --- | --- | --- | --- |
| 1 | The use terms “HIV positive men” is stigmatising by WHO, please change and use terms like “men living with HIV” or “people living with HIV” or “patients living with HIV” throughout the manuscript. | Dear reviewer, this has been rectified as per your guidance to “men living with HIV.” | Lines 31, 40, 76, 94, 185, 192, 202, 262, 309, 440, |
| 2 | Lines 75-76: “Although women in most parts of Sub-Saharan Africa (SSA), and Uganda in particular, have a higher prevalence of HIV than men”  Please use this source to support it:   - B Sartorius, JD VanderHeide, M Yang, EA Goosmann, et al. [Subnational mapping of HIV incidence and mortality among individuals aged 15–49 years in sub-Saharan Africa, 2000–18: a modelling study](https://scholar.google.com.au/citations?view_op=view_citation&hl=en&user=K7SUZ1YAAAAJ&citation_for_view=K7SUZ1YAAAAJ:L8Ckcad2t8MC). The Lancet HIV 8 (6), e363-e375. <https://www.sciencedirect.com/science/article/pii/S2352301821000515> | Dear reviewer, we appreciate this suggested resource. Unfortunately, findings from this source were not stratified by sex. It is therefore impossible to know which sex had a higher HIV prevalence |  |
| 3 | Lines 80-81: “Fewer men seek testing and counselling services on a routine basis leading to late diagnosis in late-stage disease progression”  Please use the following sources to support it:   - [The intention of men who have sex with men to participate in voluntary counseling and HIV testing and access free condoms in Indonesia](https://scholar.google.com.au/citations?view_op=view_citation&hl=en&user=K7SUZ1YAAAAJ&citation_for_view=K7SUZ1YAAAAJ:WF5omc3nYNoC). American Journal of Men's Health 12 (5), 1175-1184. <https://journals.sagepub.com/doi/full/10.1177/1557988318779737> - [Perceptions among transgender women of factors associated with the access to HIV/AIDS-related health services in Yogyakarta, Indonesia](https://scholar.google.com.au/citations?view_op=view_citation&hl=en&user=K7SUZ1YAAAAJ&citation_for_view=K7SUZ1YAAAAJ:UebtZRa9Y70C). PloS one 14 (8), e0221013. <https://journals.plos.org/plosone/article?id=10.1371/journal.pone.0221013> - [Risk Factors and the Impact of HIV among Women Living with HIV and Their Families in Yogyakarta and Belu District, Indonesia](https://scholar.google.com.au/citations?view_op=view_citation&hl=en&user=K7SUZ1YAAAAJ&cstart=20&pagesize=80&citation_for_view=K7SUZ1YAAAAJ:k_IJM867U9cC). (see Pages 35-54: scoping review on barriers and facilitators of the access of men living with HIV to HIV services; pages 224-247: findings on barriers and facilitators of the access of men living with HIV to HIV services). <https://flex.flinders.edu.au/file/eac1a04e-a24b-4888-ace8-50f06805069e/1/Fauk%20Thesis2022.pdf> | Dear reviewer, we appreciate these recommended sources. We have reviewed them and used the resources to support the statement as follows;  Low perceptions or knowledge about HIV/AIDS and the benefits of Voluntary Counseling and Testing (VCT) services have been reported for men’s negative attitudes to access HIV/AIDS services [7]. The provision of information can improve individuals’ HIV-related health literacy [8]. Health literacy is required in making critical health decisions to manage individuals’ health, including to seek and access appropriate health care [9]. In addition, men’s supportive social relationships with the healthcare professionals, their ease of access to the HIV care services, and their ability to seek the services have been reported to determine their access to the services [10]. | Lines 82-89 |
| 4 | Lines 82-83: Due to late presentation, men develop opportunistic infections with poor treatment outcomes [7] and high healthcare costs [REF].  Please use the following source to support ‘high healthcare costs:   - [Risk Factors and the Impact of HIV among Women Living with HIV and Their Families in Yogyakarta and Belu District, Indonesia](https://scholar.google.com.au/citations?view_op=view_citation&hl=en&user=K7SUZ1YAAAAJ&cstart=20&pagesize=80&citation_for_view=K7SUZ1YAAAAJ:k_IJM867U9cC). ( see Pages 35-54: scoping review on barriers and facilitators of the access of men living with HIV to HIV services; pages 224-247: findings on barriers and facilitators of the access of men living with HIV to HIV services). <https://flex.flinders.edu.au/file/eac1a04e-a24b-4888-ace8-50f06805069e/1/Fauk%20Thesis2022.pdf> | Dear reviewer, we appreciate these recommended sources. We have reviewed them and used the resources to support the statement as follows;  The costs of medical treatment, such as the costs for standard blood, viral load and CD4 tests prior to the initiation of treatment, the treatment of opportunistic infections and administration have also been revealed to be barriers to the access of HIV care services among People Living with HIV (PLHIV) [10, 12, 13, 14] | Lines 90-94 |
|  | **Data management and analysis** |  |  |
| 5 | Are the field researchers (research assistants) the authors in the paper? If yes, please put their initials to make clear who did what. Who are the three people in the analysis team? Please provide their initials in bracket. | Dear reviewer, the research assistants are not part of the authors. They were recruited, and trained to do data collection.  The analysis team comprised of three people (S.N, W.A, J.T) | Line 229 |
| 6 | I suggest the authors use the following resources to support their description of data analysis:   - Cultural and religious determinants of HIV transmission: A qualitative study with people living with HIV in Belu and Yogyakarta, Indonesia. PLoS ONE 16(11): e0257906. <https://journals.plos.org/plosone/article?id=10.1371/journal.pone.0257906> - Qualitative Data Analysis for Applied Policy Research. In: Bryman A, Burgess RG, editors. London: Routledge; 1994. p. 173–94. | We appreciate this suggestion. These resources have supported us to strengthen the description of our data analysis as follows;  Data analysis was guided by the five steps of qualitative data analysis framework [28]. These include (i) familiarization with the data by reading through transcripts, listening to the audios and going through the field notes; (ii) identification of a thematic framework by making notes, writing down recurrent themes and issues which emerge as important to study respondents; (iii) indexing the data by applying the thematic framework to the individual transcripts, grouping similar codes under the same themes and sub-themes; (iv) charting data through arranging appropriate thematic references in a summary of chart; and (v) mapping and interpretation of the data through which data were examined and interpreted [28-29]. | Lines 243- 251 |
|  | **Discussion** |  |  |
| 7 | Lines 389-390: “Internalised stigma has been found to impact certain decisions like avoiding to seek care when needed.”  Please use the following resources to support it:   - HIV Stigma and Moral Judgement: Qualitative Exploration of the Experiences of HIV Stigma and Discrimination among Married Men Living with HIV in Yogyakarta. *Int. J. Environ. Res. Public Health* **2020**, *17*, 636. <https://doi.org/10.3390/ijerph17020636>. - [HIV stigma and discrimination: perspectives and personal experiences of healthcare providers in Yogyakarta and Belu, Indonesia](https://scholar.google.com.au/citations?view_op=view_citation&hl=en&user=K7SUZ1YAAAAJ&citation_for_view=K7SUZ1YAAAAJ:4DMP91E08xMC). *Frontiers in medicine* 8, 625787. <https://www.frontiersin.org/articles/10.3389/fmed.2021.625787/full> | Dear reviewer, we appreciate this suggestion. These resources have supported us to strengthen the statement as follows;  A study in Indonesia reported that participants’ individual moral judgement that associated their HIV status with amoral behaviours and engagement in sex with female sex workers, and participants’ self-judgement as ‘dirty people’ or sinners which increased guilty feelings, were additional factors leading to HIV’s perceived stigma [37] | Lines 423-427 |
| 8 | Lines 394-396: “On the other hand, “enacted stigma,” which is the lived experience of discrimination against an HIV positive person through others’ actions or words, is also known to have a negative impact on health outcomes.”  Please use the following resources to support it:   - HIV Stigma and Moral Judgement: Qualitative Exploration of the Experiences of HIV Stigma and Discrimination among Married Men Living with HIV in Yogyakarta. *Int. J. Environ. Res. Public Health* **2020**, *17*, 636. <https://doi.org/10.3390/ijerph17020636>. - Stigma and Discrimination towards People Living with HIV in the Context of Families, Communities, and Healthcare Settings: A Qualitative Study in Indonesia. *Int. J. Environ. Res. Public Health* **2021**, *18*, 5424. <https://doi.org/10.3390/ijerph18105424> | Dear reviewer, we appreciate this suggestion. These resources have supported us to strengthen the statement as follows;  Individuals have revealed having experienced HIV-related discrimination attitudes from healthcare professionals within healthcare settings. For example, in the Indonesia study, it was reported that clients were left untreated for many days in healthcare facilities [37]. HIV stigma and discrimination by healthcare professionals traumatized the PLHIV, thus concealment of HIV status, hindering access to healthcare services [37, 39, 40] thereby influencing their health and wellbeing in the long run. | Lines 429-435 |

Sincerely,

Sherifah Nabikande

Corresponding Author
